# Supplementary material for: Pressure dependence in aqueous-based electrochemical CO2 reduction
Source: Nat Commun. 2023 May 23;14:2958. doi: 10.1038/s41467-023-38775-0 (PMC10205702; doi:10.1038/s41467-023-38775-0)
Supplement: Supplementary file 1 — Supplementary Information [file 41467_2023_38775_MOESM1_ESM.pdf]

Supplementary Information for

Pressure dependence in aqueous-based electrochemical CO_2 reduction

Liang Huang, Ge Gao, Chaobo Yang, Xiao-Yan Li, Rui Kai Miao, Yanrong Xue, Ke Xie, Pengfei Ou, Cafer T. Yavuz, Yu Han, Gaetano Magnotti,† David Sinton,† Edward H. Sargent,† Xu Lu†

Correspondence to: Email: Xu Lu (xu.lu@kaust.edu.sa); Edward H. Sargent (ted.sargent@utoronto.ca); David Sinton (sinton@mie.utoronto.ca); Gaetano Magnotti (gaetano.magnotti@kaust.edu.sa)

This PDF file includes:

Supplementary Figs. 1 to 22

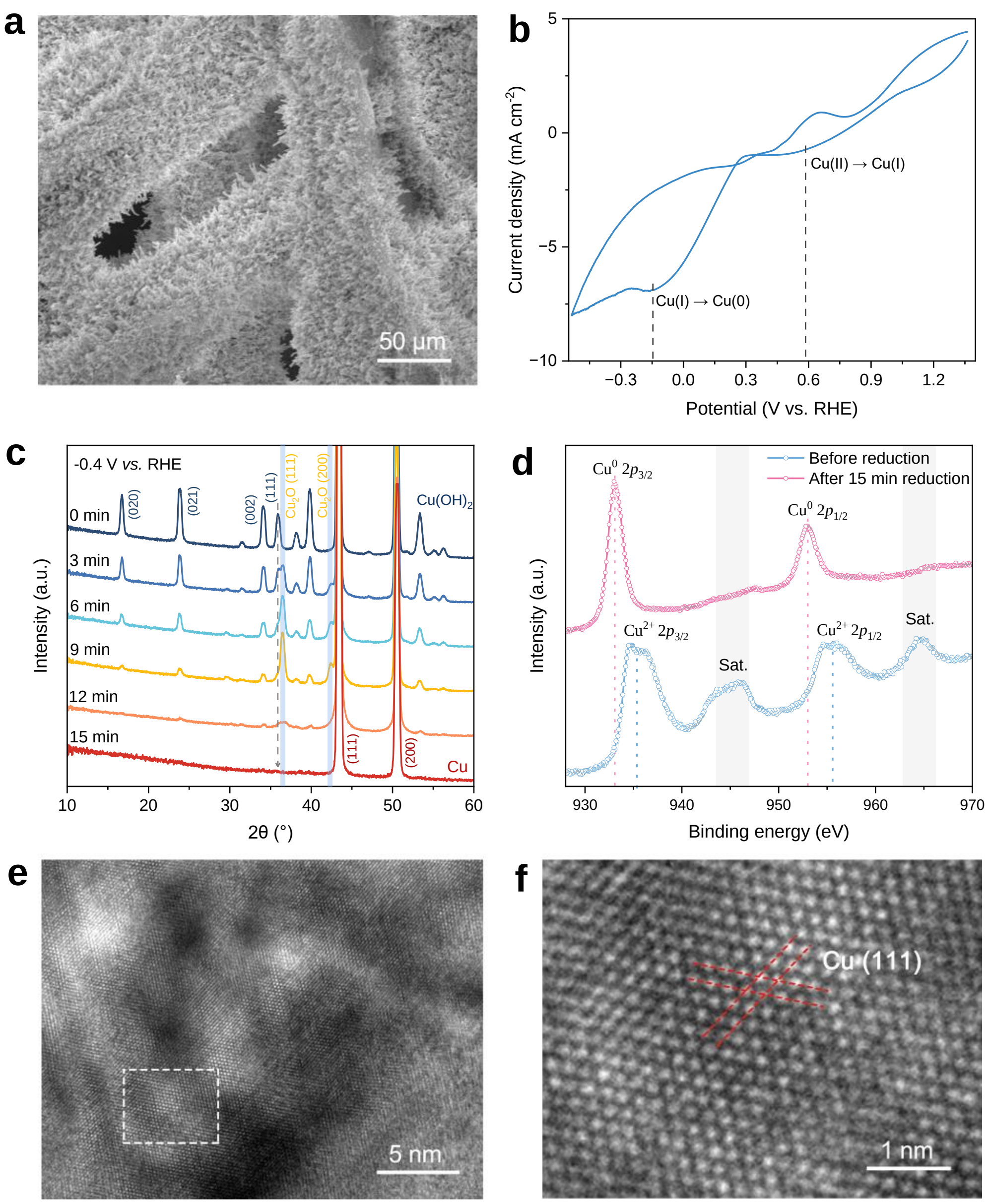

Supplementary Fig. 1. (a) SEM image of the as-prepared Cu catalyst. (b) Cyclic voltammogram of $Cu(OH)_2$ in Ar-saturated 0.5 M $KHCO_3$ aqueous solution with a scan rate of 50 mV s^{-1}. Two reduction peaks can be observed, corresponding to the Cu(II) → Cu(I) → Cu(0) process under reduction conditions. (c) Time-resolved *operando* XRD patterns of the catalyst showing its transition from $Cu(OH)_2$ to Cu at -0.4 V vs. RHE. (d) Cu 2*p* XPS spectra of the catalyst before and after the 15 min electroreduction in (c), confirming the transition from $Cu(OH)_2$ to Cu. (e) TEM image of the as-prepared Cu catalyst, indicating that the Cu crystals are highly crystalline. (f) Enlarged HRTEM image of the square-enclosed area in (e).

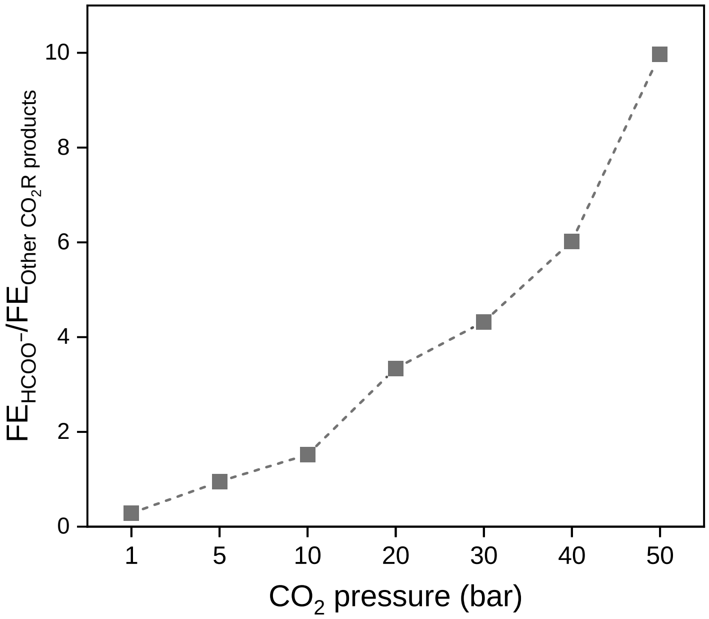

Supplementary Fig. 2. FE ratios of formate to other CO_2R products on the Cu catalyst under different pressures at -1.1 V vs. RHE.

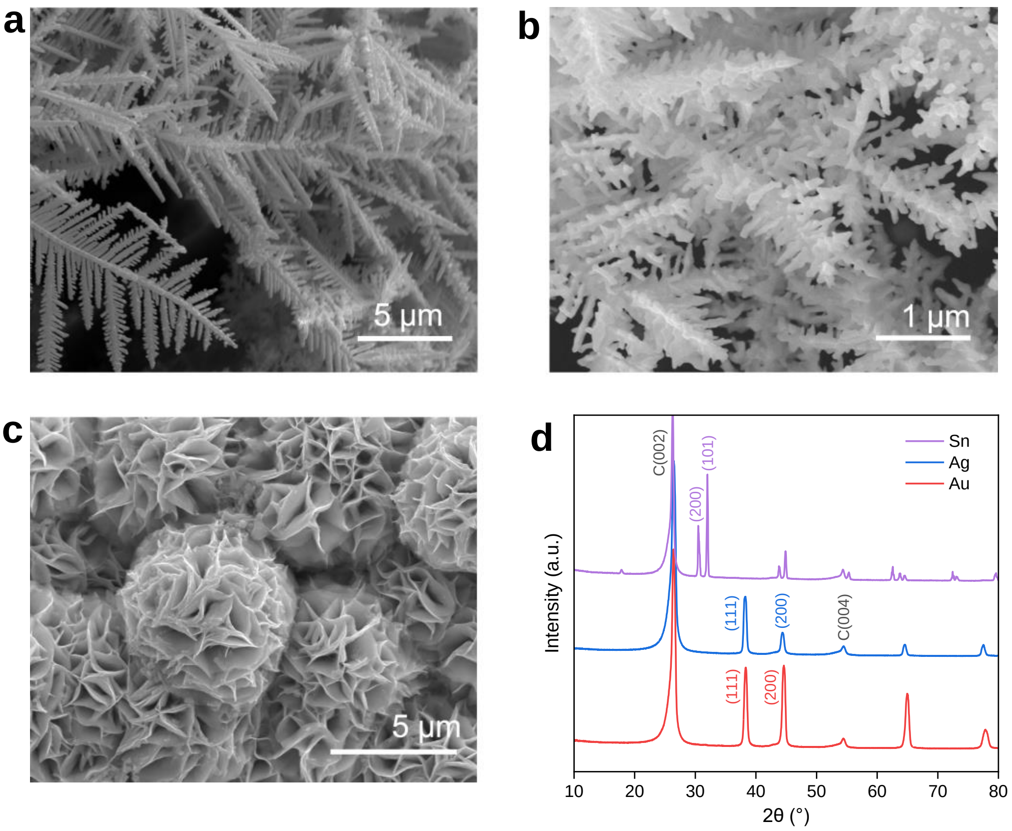

Supplementary Fig. 3. SEM images of the as-prepared (a) Au, (b) Ag, and (c) Sn catalysts. (d) XRD patterns of the as-prepared Au, Ag, and Sn catalysts.

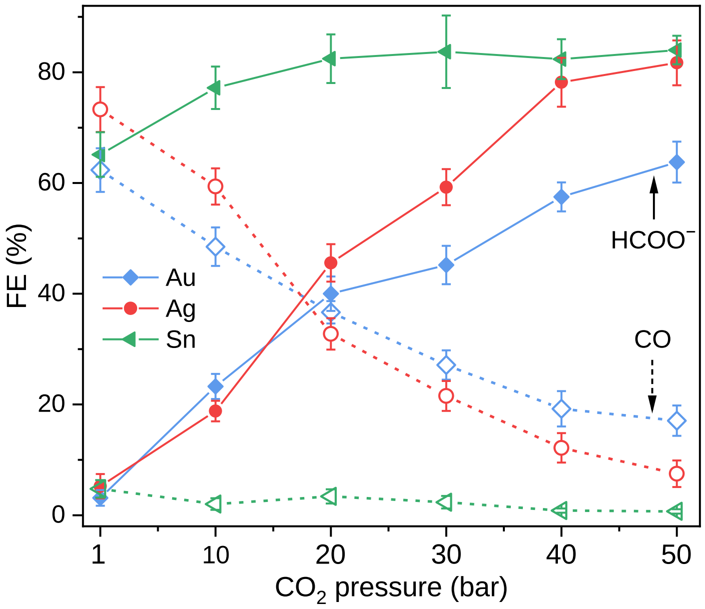

Supplementary Fig. 4. FEs toward formate and CO on Au, Ag, and Sn catalysts under different pressures at -1.1 V vs. RHE.

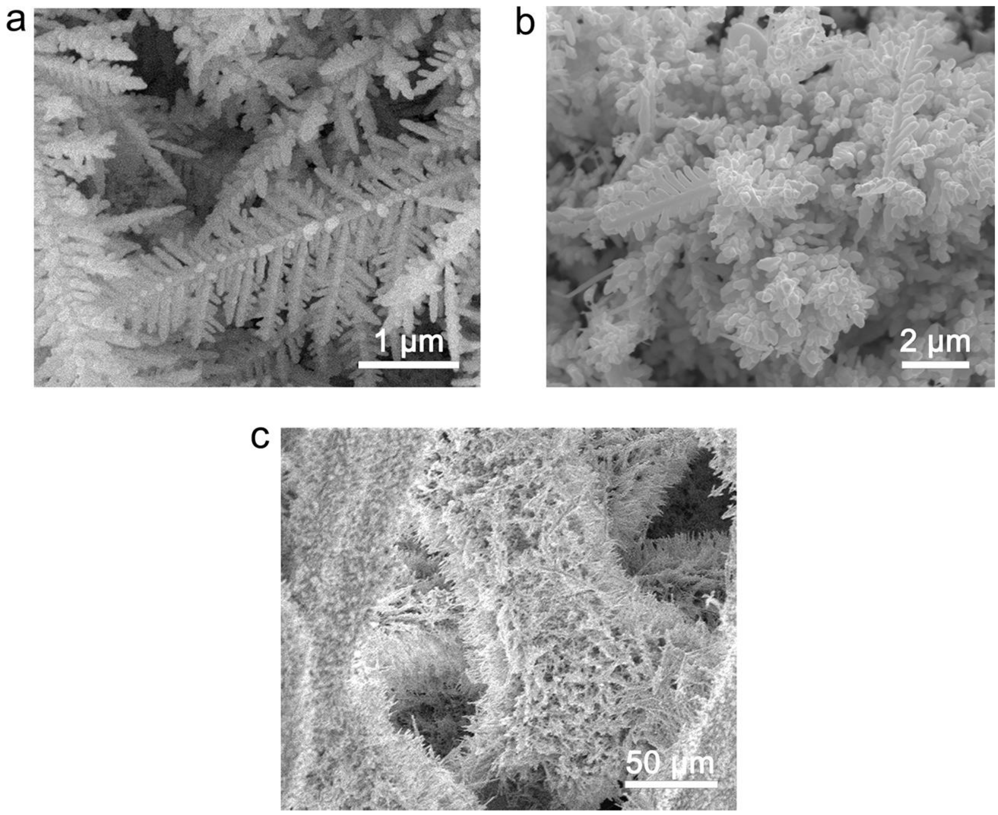

Supplementary Fig. 5. SEM images of (a) Au, (b) Ag, and (c) Cu catalysts after 1 h CO_2 electrolysis at -1.1 V vs. RHE under 50 bar.

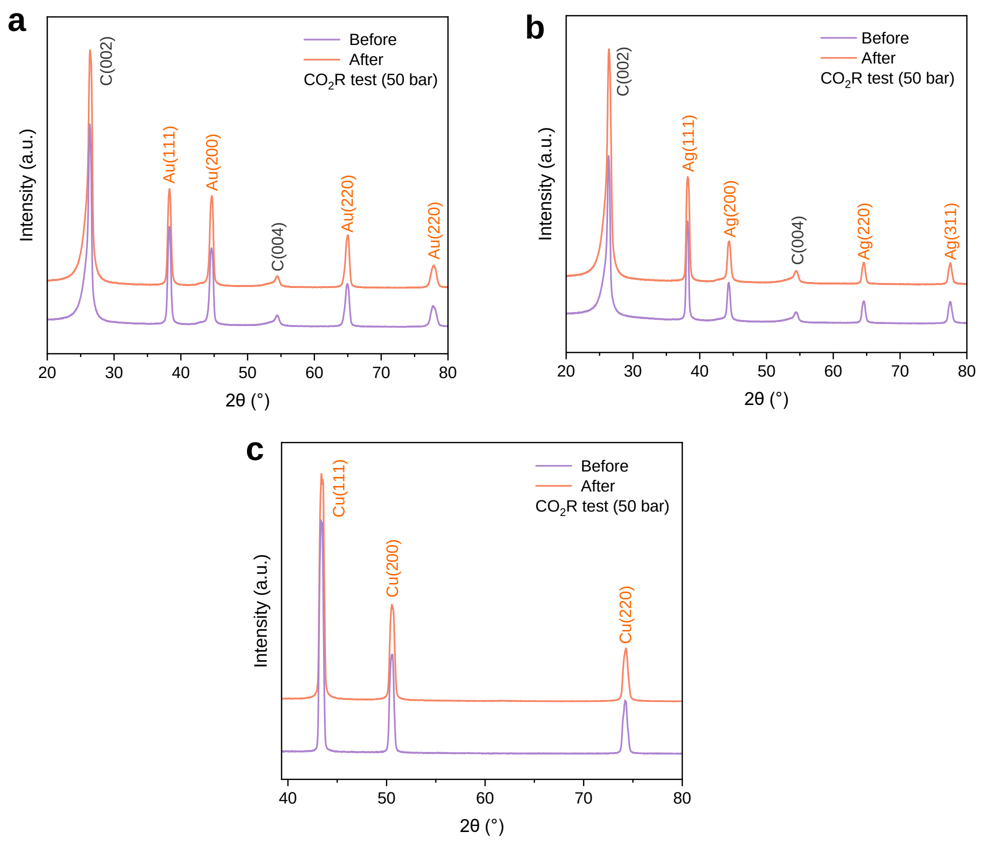

Supplementary Fig. 6. XRD patterns of (a) Au, (b) Ag, and (c) Cu catalysts before and after 1 h CO_2 electrolysis at -1.1 V vs. RHE under 50 bar.

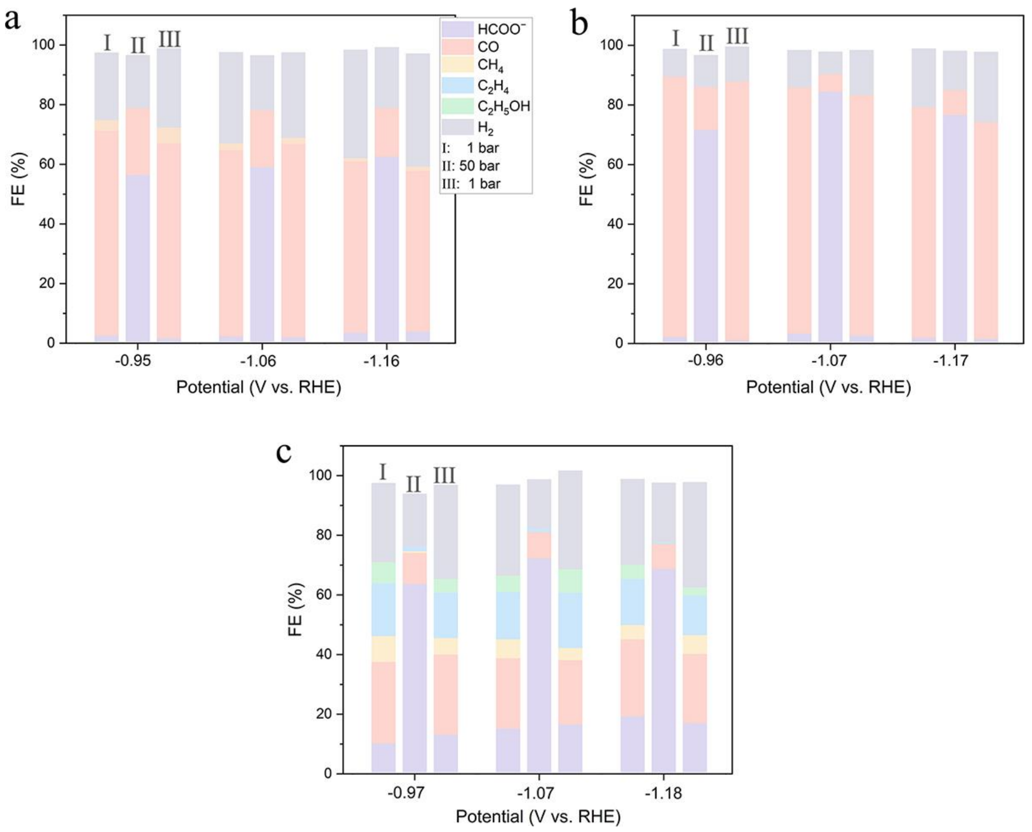

Supplementary Fig. 7. FEs toward CO_2R products and H_2 on (a) Au, (b) Ag, and (c) Cu catalysts that were successively operated under 1 bar, 50 bar, and 1 bar at cathode potentials ranging from -0.95 to -1.18 V vs. RHE.

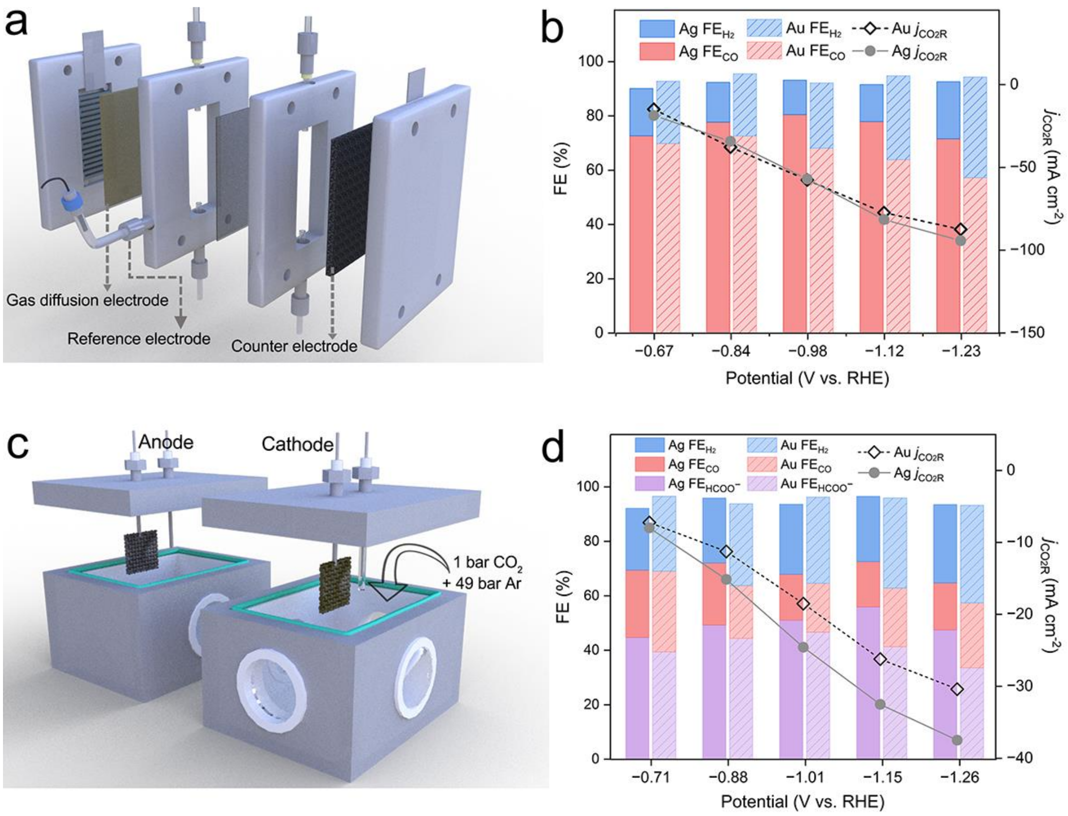

Supplementary Fig. 8. (a) Schematic of the ambient-pressure gas-fed flow cell. (b) FEs toward CO and H_2, and CO_2R partial current densities on the Au and Ag catalysts in the flow cell described in (a). (c) Schematic of the high-pressure H-cell filled with 50 bar CO_2/Ar mixture (1 bar CO_2 and 49 bar Ar). (d) FEs toward formate, CO and H_2, and CO_2R partial current densities on the Au and Ag catalysts in the H-cell described in (c).

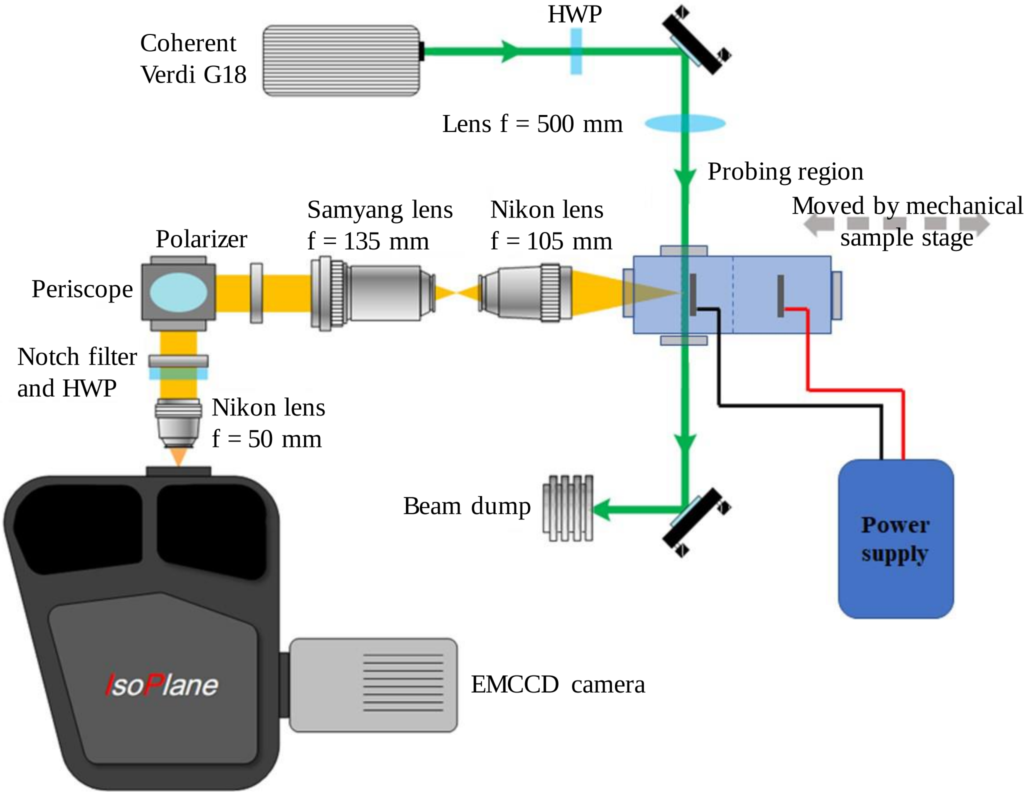

Supplementary Fig. 9. Schematic of the custom-built *operando* Raman system with the high-pressure H-cell.

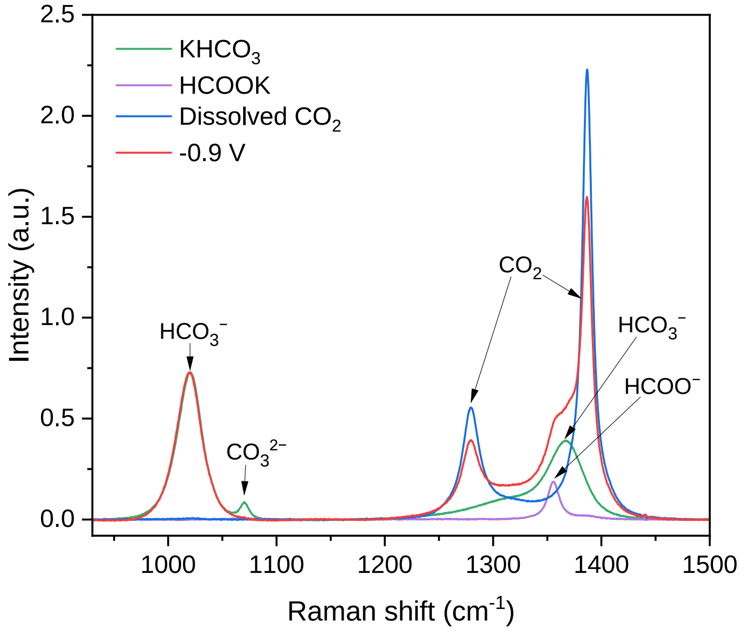

Supplementary Fig. 10. *Operando* Raman spectrum acquired on the Cu surface at -0.9 V vs. RHE under 50 bar, and the Raman spectra of $KHCO_3$ (0.5 M), HCOOK (0.05 M), and dissolved CO_2 (50 bar) in aqueous solutions.

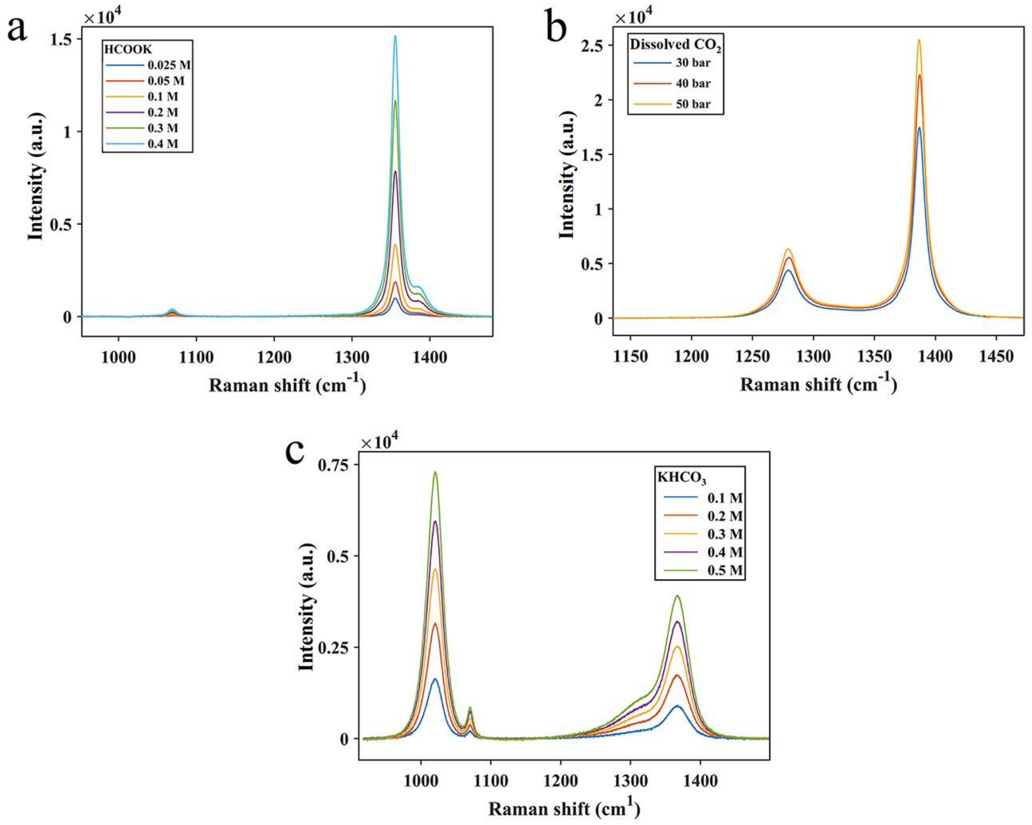

Supplementary Fig. 11. Raman spectra of (a) HCOOK, (b) dissolved CO_2, and (c) $KHCO_3$ aqueous solutions with various concentrations.

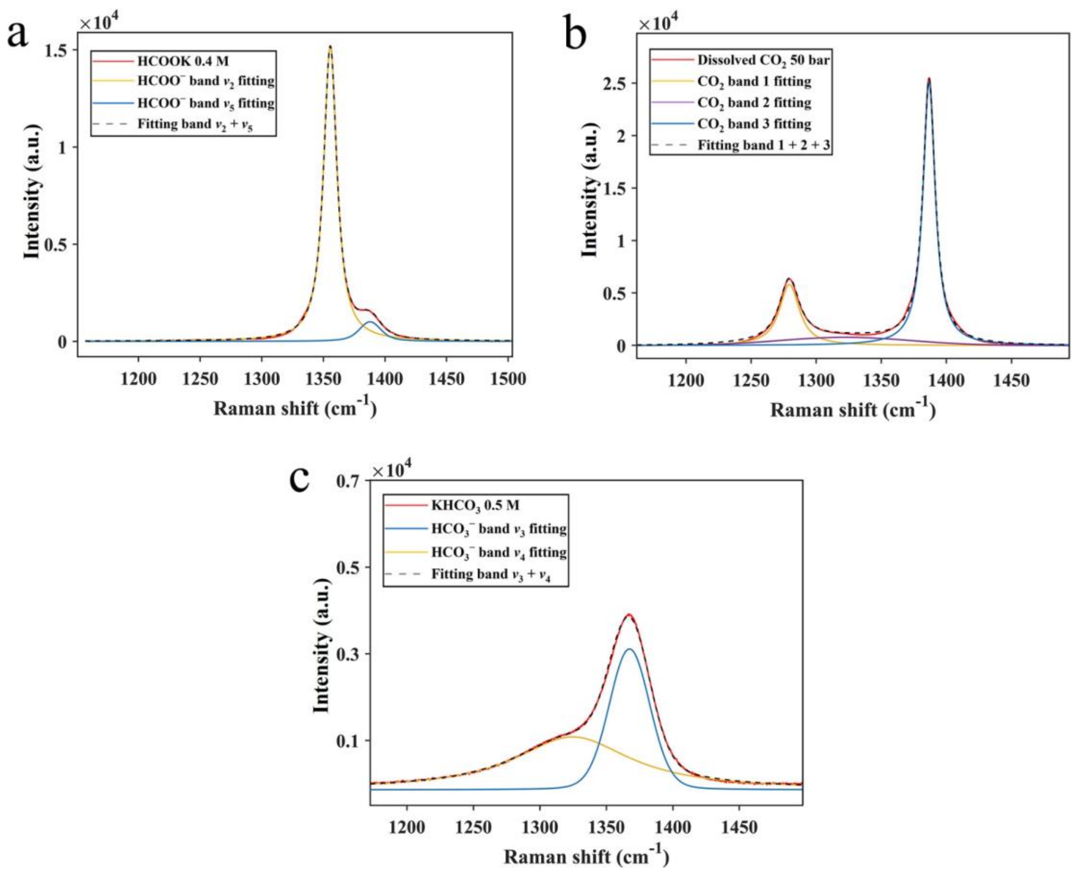

Supplementary Fig. 12. Band assignments and fitting curves for (a) HCOOK, (b) dissolved CO_2, and (c) $KHCO_3$ aqueous solutions with selected concentrations.

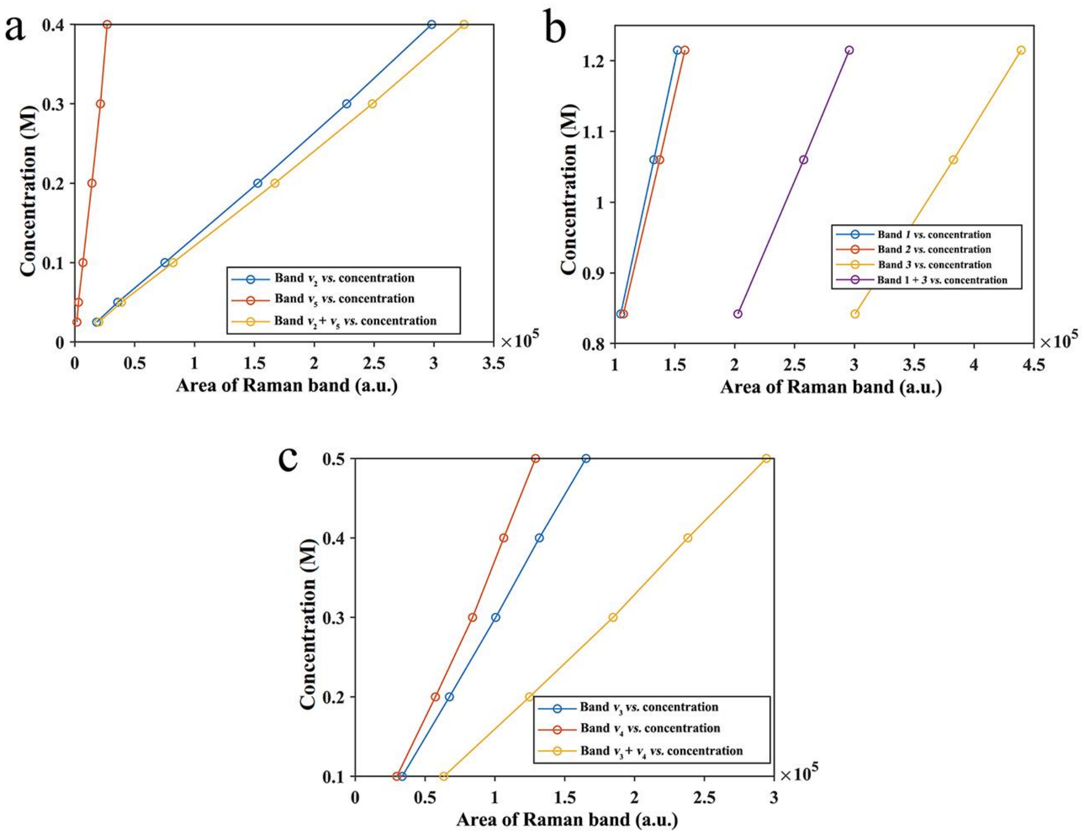

Supplementary Fig. 13. Calibration curves for (a) $HCOO^-$, (b) dissolved CO_2, and (c) HCO_3^-.

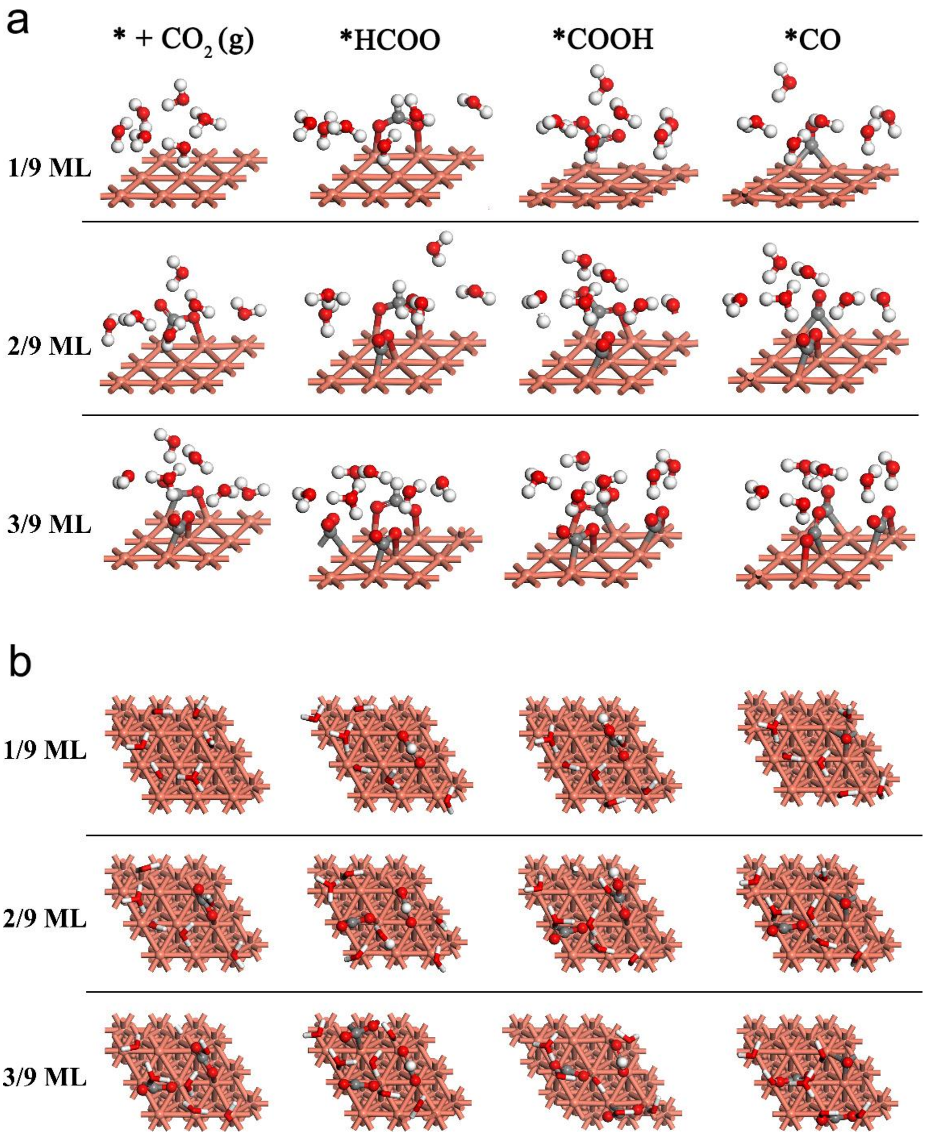

Supplementary Fig. 14. Optimized structures of adsorbed CO_2R intermediates for HCOOH and *CO production with the CO_2 coverages of 1/9 ML, 2/9 ML, and 3/9 ML. (a) Side view. The bottom three Cu atomic layers were not shown for clear demonstration. (b) Top view. The water layers were drawn sticks to clearly show the intermediates. Cu: orange; O: red; C: gray; H: white.

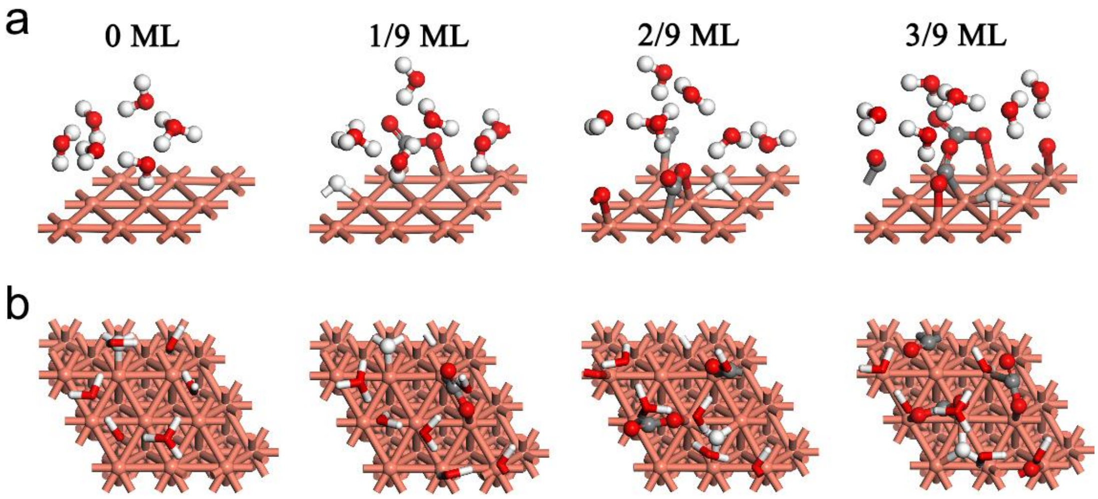

Supplementary Fig. 15. Optimized structures of *H for HER with the CO_2 coverages of 0 ML, 1/9 ML, 2/9 ML, and 3/9 ML. (a) Side view. The bottom three Cu atomic layers were not shown for clear demonstration. (b) Top view. The water layers were drawn sticks to clearly show the intermediates. Cu: orange; O: red; C: gray; H: white.

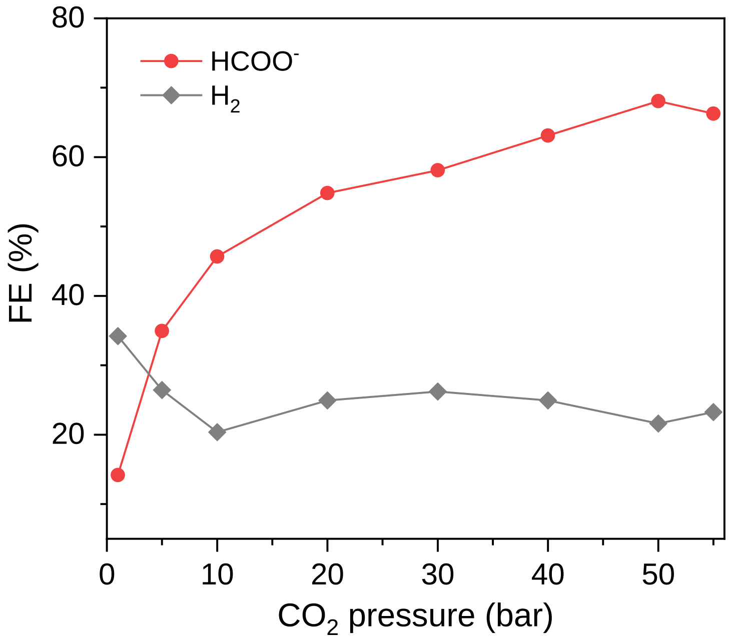

Supplementary Fig. 16. FEs of formate and H_2 on the Cu catalyst under different pressures at -1.1 V vs. RHE. The HER selectivity was insignificantly affected by pressure.

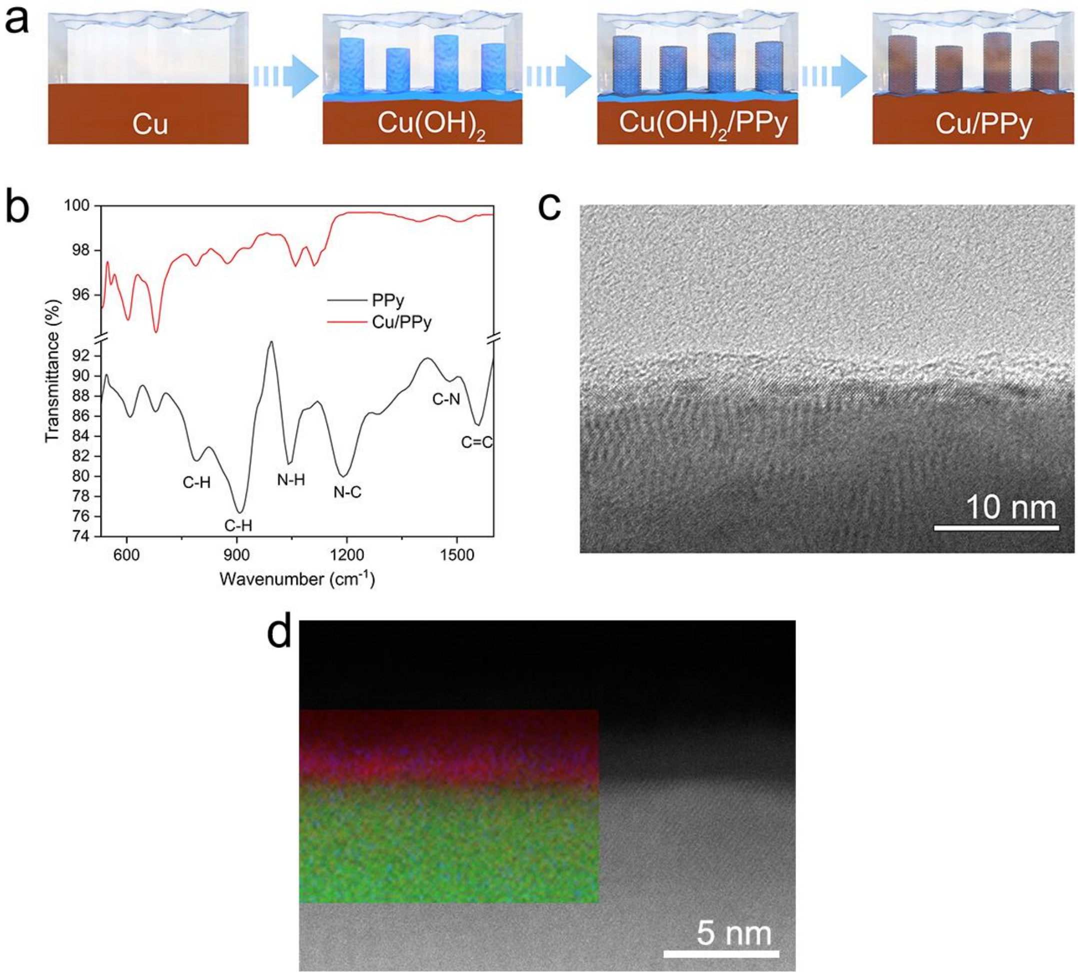

Supplementary Fig. 17. (a) Schematic illustration of the preparation of the Cu/PPy catalyst. (b) FTIR spectra of Cu/PPy and PPy. (c) TEM and (d) STEM images of the Cu/PPy catalyst. Inset in (d) depicted the overlaid EELS mapping of the selected area. Green, Cu; red, C; blue, N.

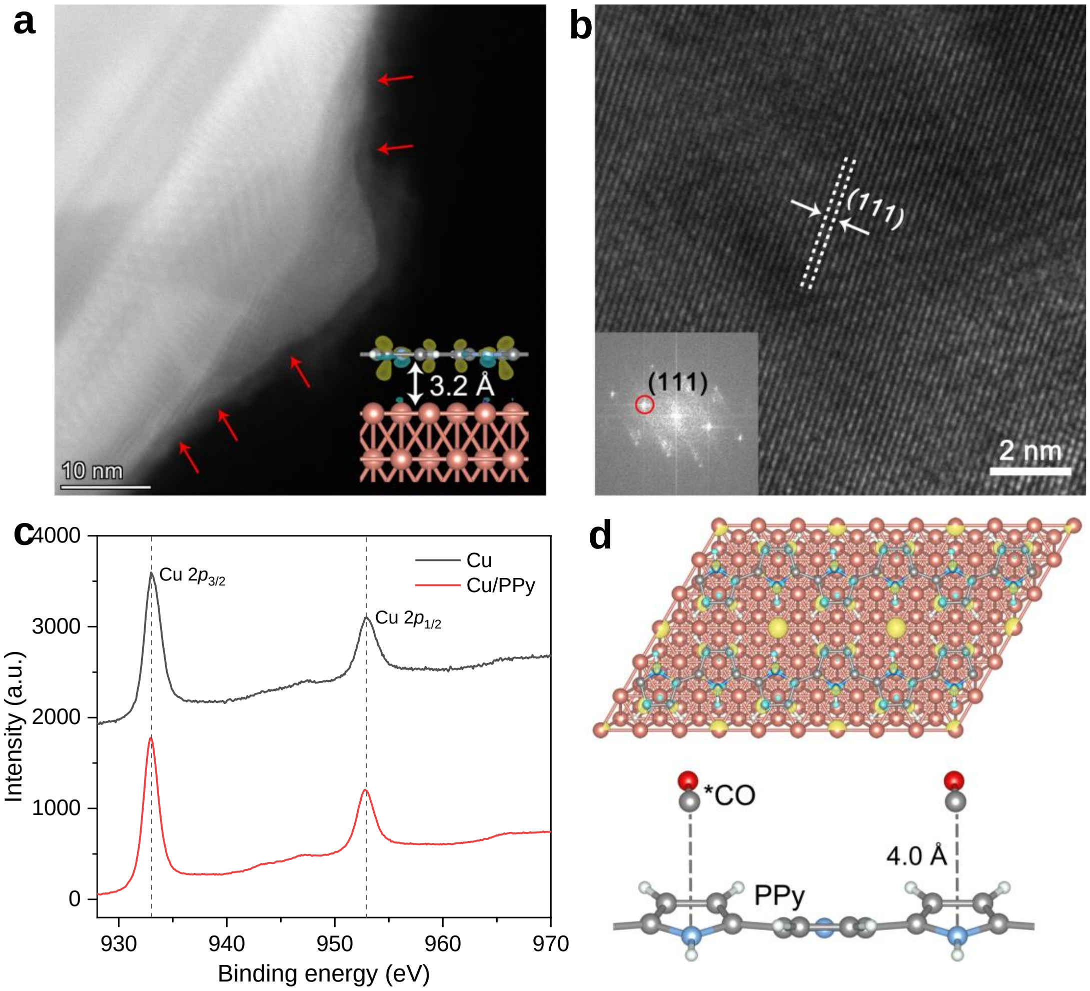

Supplementary Fig. 18. (a) STEM image of the Cu/PPy catalyst. The inset showed the simulated interlayer spacing between the Cu(111) facet and PPy layer. The red arrows indicated the areas with loose PPy or naked Cu sites. (b) HRTEM image of the Cu/PPy nanoparticles. The inset was the corresponding fast Fourier transform (FFT) pattern. (c) Cu 2*p* XPS spectra of the Cu and Cu/PPy catalysts. (d) The charge density difference for the Cu/PPy catalyst. The yellow and cyan colors indicated the charge accumulation and depletion, respectively. (e) The optimized structure of adsorbed intermediates on the PPy layer. Cu: orange; N: blue; O: red; C: gray; H: white.

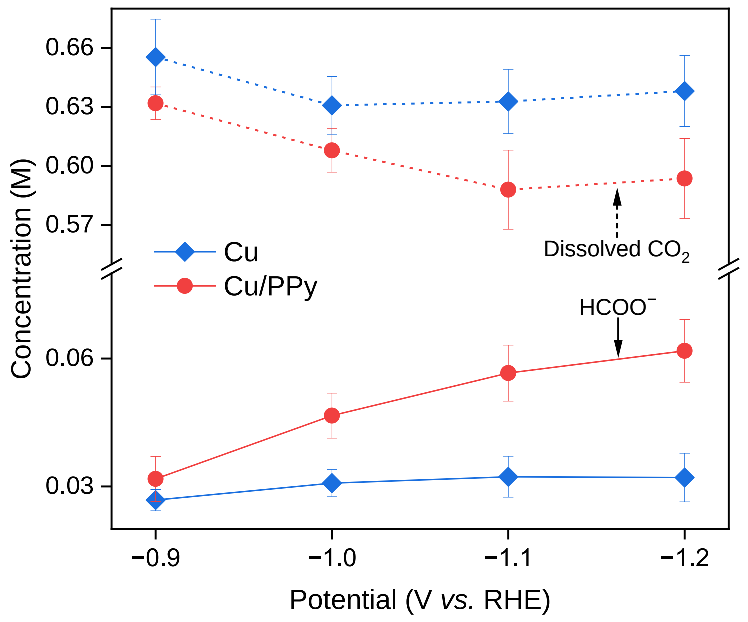

Supplementary Fig. 19. Measured concentrations of dissolved CO_2 (dashed) and $HCOO^-$ (solid) on the surface of Cu and Cu/PPy cathodes (x = 0 μm) against the cathode potential under 50 bar. Error bars represent the standard deviation of thirty consecutive measurements.

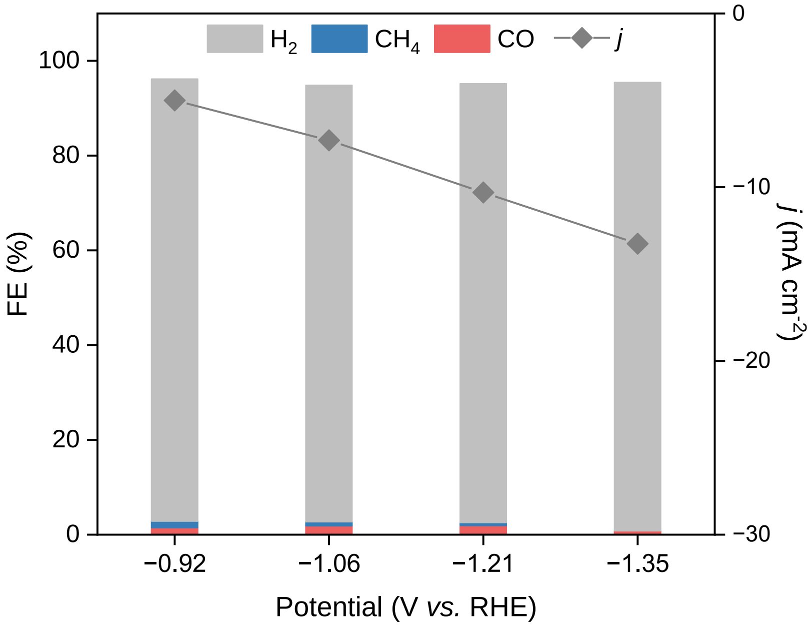

Supplementary Fig. 20. CO_2R performance on bare PPy in an ambient-pressure H-cell filled with CO_2 saturated 0.5 M $KHCO_3$ aqueous solution.

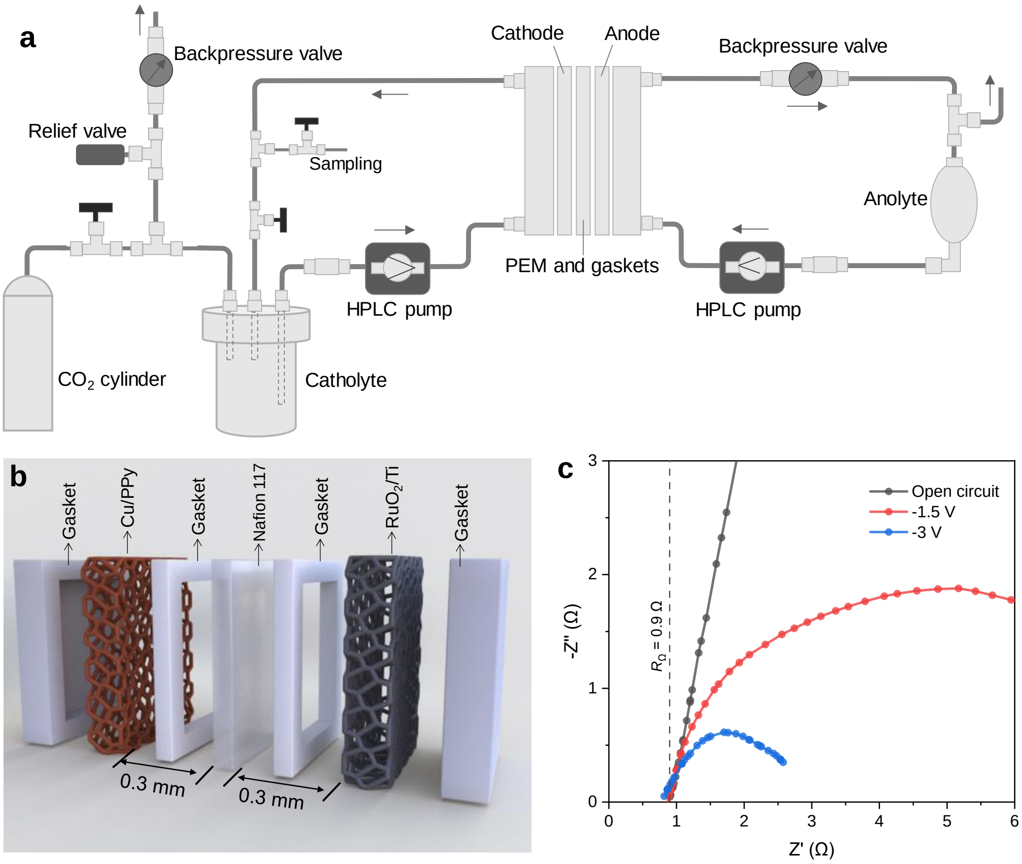

Supplementary Fig. 21. (a) Flow diagram and (b) schematic of the high-pressure narrow-gap aqueous flow cell system. (c) Electrochemical impedance spectroscopy (EIS) of the high-pressure narrow-gap aqueous flow cell.

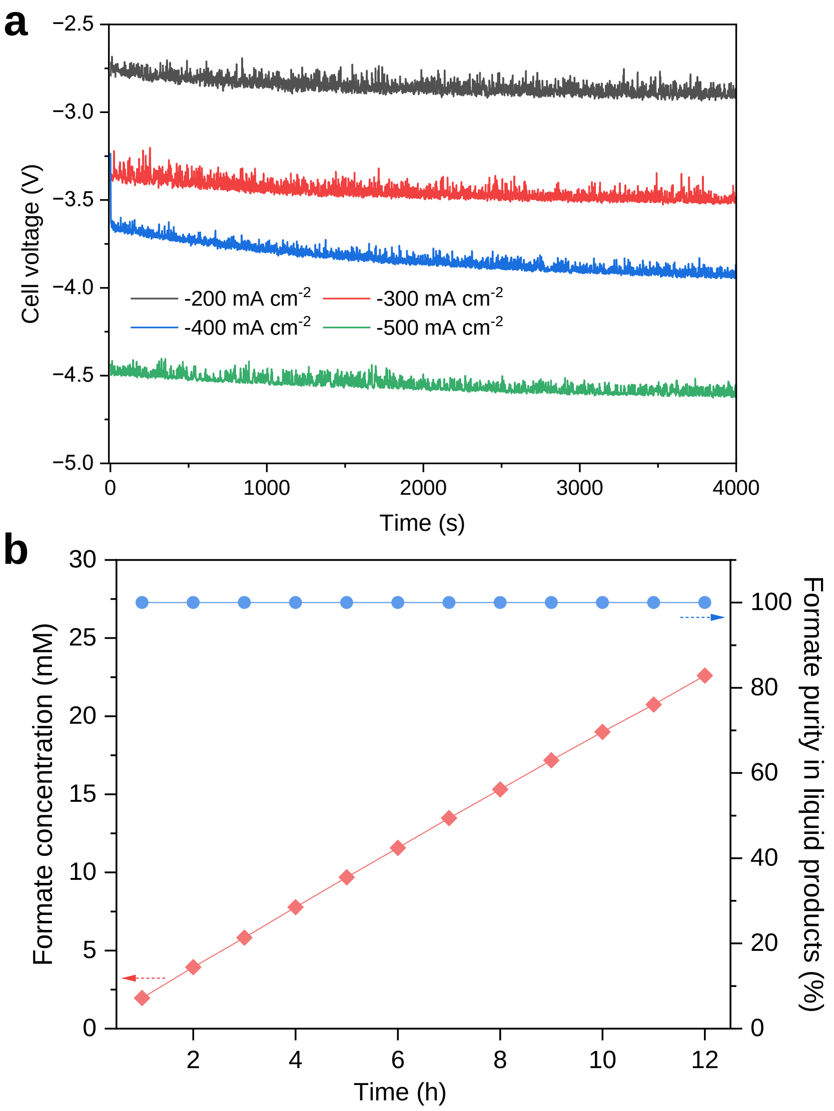

Supplementary Fig. 22. (a) Chronopotentiograms at total current densities of 200, 300, 400 and 500 mA cm^{-2} on Cu/PPy in the high-pressure narrow-gap aqueous flow cell fed by 50 bar PCO_2. (b) Formate concentration and relative purity over 12 h continuous CO_2R in the narrow-gap aqueous flow cell at a current density of 400 mA cm^{-2} under 50 bar.
